# The Forty-third Annual Session of the Medical Association of Georgia

**Published:** 1892-05

**Authors:** 


### THE FORTY-THIRD ANNUAL SESSION OF THE MEDICAL ASSOCIATION OF GEORGIA.

---

This meeting was held at Columbus on the 20th, 21st and 22d of April. Officers were: President, G. W. Mulligan, M. D., Washington; Vice-Presidents, James M. Hull, M. D., Augusta, Mark O'Daniel, M. D., Macon. Secretary, Dan. H. Howell, M. D., Atlanta; Treasurer, E. C. Goodrich, M. D., Augusta.

*Board of Censors*—B. R. Doster, M. D., Blakely; G. W. Mulligan, M. D., Washington; H. McHatton, M. D., Macon; Eugene Foster, M. D., Augusta; K. P. Moore, M. D., Macon.

*Chairman Committee of Arrangements.*—George J. Grimes, M. D., Columbus.

*Chairman Committee on Programme.*—J. F. Lancaster, M. D., Forsyth.

*Chairman on New Membership.*—E. C. Goodrich, M. D., Augusta.

The meeting was quite successful and largely attended. Nearly half the counties in the State were represented by one or more members.

Many interesting and instructive papers were read before the Association, but we noted the conspicuous absence of many that were promised on the preliminary programme.

Columbus threw open her doors to the visitors and made a successful effort to make the members of the Association have a thoroughly enjoyable meeting. The banquet given at the Rankin House, on the evening of the 21st, by the resident physicians and the citizens of Columbus was thoroughly enjoyed and appreciated. The responses to toasts by Drs. McIntosh, Westmoreland, Griggs, Jordan, Ridley of LaGrange, Gaston, ex-representative Chappell, lawyer Lionel Levy and Mayor Slade, were eloquent and appropriate. Chairman Grimes acted toast-master in an easy and graceful manner. On behalf of appreciative members of the Association, Dr. Westmoreland presented Dr. Grimes with an elegant umbrella; and Dr. Howell with a match box.

During the session of the Association a telegram of sympathy in his illness was sent to Dr. J. S. Todd, of Atlanta.

Officers elected for the ensuing year were as follows: President, Dr. A. A. Smith, Hawkinsville; Vice-Presidents, Drs. George J. Grimes, Columbus; Dr. Robert Taylor, Griffin; Treasurer, Dr. E. C. Goodrich, Augusta.

TO VACANCIES ON THE BOARD OF CENSORS.—Dr. Hopkins, of Thomasville, and Dr. O'Daniel, of Atlanta.

Americus was selected as the place of next year's meeting. Everybody was charmed with Columbus and filled with admiration at her prosperity. Her factories, wholesale houses, hotels, steamboats, railroads, horse, electric and dummy cars show her to be a hustling city, fully able to take care of herself. We regret that space will not permit us to mention by name the various physicians to whom THE JOURNAL is indebted for favors.

Our friends, Daniel and Phillips, had excellent exhibits of instruments in the vestibule of the opera house and seem to be doing a nice business in their goods. We thank Mr. Perryman for special favors. In the Rankin House, Sharp & Dohme, Doliber, Goodale Co., The Maltine Co., Jno. Wyeth & Bro., Angier Chemical Co., The Nedofik Co., E. Fongera & Co., Fairchild Bros. & Foster, N. Y. Pharmacal Association, and possiby others, had exhibits.

We hope the meeting at Americus will be even more largely attended than was that at Columbus, and further, that a good deal of harmony and a very little of politics will be present.

